# Genetic risk for PCOS/PMOS is associated with hyperandrogenism but not irregular menses in adolescents and young adults

**DOI:** 10.1210/jendso/bvag204

**Published:** 2026-09-01

**Authors:** Varun Lingadal, Mali DiMeo, Joel N Hirschhorn, Yee-Ming Chan, Jia Zhu

**Affiliations:** Division of Endocrinology, Department of Pediatrics, Boston Children's Hospital, Boston, MA 02115, USA; Programs in Metabolism and Medical and Population Genetics, The Broad Institute of MIT and Harvard, Cambridge, MA 02142, USA; Division of Endocrinology, Department of Pediatrics, Boston Children's Hospital, Boston, MA 02115, USA; Programs in Metabolism and Medical and Population Genetics, The Broad Institute of MIT and Harvard, Cambridge, MA 02142, USA; Division of Endocrinology, Department of Pediatrics, Boston Children's Hospital, Boston, MA 02115, USA; Programs in Metabolism and Medical and Population Genetics, The Broad Institute of MIT and Harvard, Cambridge, MA 02142, USA; Department of Pediatrics, Harvard Medical School, Boston, MA 02115, USA; Department of Genetics, Harvard Medical School, Boston, MA 02115, USA; Division of Endocrinology, Department of Pediatrics, Boston Children's Hospital, Boston, MA 02115, USA; Programs in Metabolism and Medical and Population Genetics, The Broad Institute of MIT and Harvard, Cambridge, MA 02142, USA; Department of Pediatrics, Harvard Medical School, Boston, MA 02115, USA; Division of Endocrinology, Department of Pediatrics, Boston Children's Hospital, Boston, MA 02115, USA; Programs in Metabolism and Medical and Population Genetics, The Broad Institute of MIT and Harvard, Cambridge, MA 02142, USA; Department of Pediatrics, Harvard Medical School, Boston, MA 02115, USA

**Keywords:** polycystic ovary syndrome, PCOS, polyendocrine metabolic ovarian syndrome, PMOS, at risk for PMOS, ALSPAC

## Abstract

**Context:**

Diagnosing polycystic ovary syndrome, recently renamed polyendocrine metabolic ovarian syndrome (PMOS), during adolescence is challenging because menstrual irregularity and clinical signs suggesting hyperandrogenism are common. Recent international recommendations highlighted an “at risk for PMOS” category, defined by either menstrual regularity or hyperandrogenism, to guide surveillance; however, its prevalence and genetic correlates remain unknown.

**Objective:**

To estimate the prevalence of PMOS and “at risk for PMOS” in adolescence and young adulthood and to assess associations with genetic risk for PMOS.

**Design/Setting/Participants:**

Retrospective analysis of 1533 participants from the ALSPAC study, a UK birth cohort, with sufficient data to classify menstrual regularity and hyperandrogenism, and a subset of 1371 genotyped participants of European ancestry.

**Main Outcome Measures:**

Prevalence of PMOS and “at risk for PMOS” and associations with a PMOS polygenic score (PGS).

**Results:**

Among 1533 participants, 49 (3%) met criteria for PMOS and 417 (27%) were “at risk for PMOS” (hyperandrogenism only, 276 [18%]; irregular menses only, 141 [9%]). A higher PMOS PGS was associated with hyperandrogenism only (OR per SD: 1.22; 95% CI: 1.07-1.39; *P* = 4 × 10^−3^) but not with irregular menses only (OR: 0.98; 95% CI: 0.83-1.15; *P* = .8). The association with PMOS was positive but not statistically significant (OR: 1.28; 95% CI: 0.88-1.87; *P* = .2).

**Conclusion:**

PMOS prevalence in adolescence and young adulthood was 3%, whereas over one-quarter met criteria for being “at risk for PMOS.” Genetic risk for PMOS was associated with hyperandrogenism but not isolated menstrual irregularity, suggesting androgen excess may represent a more specific early manifestation of inherited PMOS liability.

Polyendocrine metabolic ovarian syndrome (PMOS), recently renamed from polycystic ovary syndrome is a common endocrine disorder that often manifests during adolescence and young adulthood and carries lifelong implications for reproductive and cardiometabolic health (1-4). Early identification, however, remains clinically challenging because the defining features of PMOS, menstrual irregularity and clinical signs of hyperandrogenism (eg, acne), are common in adolescent and young adult girls (5). Many may present with only one feature, either irregular menstrual cycles or clinical/biochemical features suggesting androgen excess, and may or may not develop the second feature in the future. Others can have apparent features of PMOS that are transient and resolve without intervention. As a result, it is often unclear which findings represent early manifestations of PMOS and which are unrelated. These diagnostic challenges lead to uncertainty in clinical counseling, monitoring, and early intervention for PMOS and its associated health conditions (6).

To address these challenges, the 2025 international evidence-based recommendations for PMOS in adolescents established a diagnostic framework emphasizing persistent menstrual disturbance and hyperandrogenism as the defining features of PMOS (6, 7). Importantly, these recommendations emphasize an “at risk for PMOS” category for individuals meeting only 1 diagnostic criterion, either irregular menses or hyperandrogenism, which may represent an early phenotype. Emerging evidence suggests that adolescents classified as “at risk for PMOS” already exhibit early metabolic alterations, including higher blood pressure and insulin resistance (8, 9). Therefore, adolescents and young adults within this “at risk” category may therefore warrant closer clinical surveillance. Characterizing the prevalence of this “at risk” category and its underlying genetic correlates could inform strategies to identify and monitor individuals at elevated risk for PMOS, and thereby, create opportunities for earlier intervention to prevent or delay progression toward PMOS and its related conditions.

Genome-wide association studies have identified numerous genetic variants associated with PMOS in adult women (10). Using data from the Avon Longitudinal Study of Parents and Children (ALSPAC), a population-based birth cohort with detailed reproductive phenotyping and genome-wide genotyping, we aimed to (1) estimate the prevalence of PMOS and “at risk for PMOS” classifications in adolescence and young adulthood based on current guideline criteria and (2) test whether polygenic risk for PMOS is associated with these phenotypes.

## Methods

### Study cohort

The ALSPAC is a population-based birth cohort that recruited pregnant women with expected delivery dates between April 1, 1991 and December 31, 1992. Of the initial 14 451 mother–child pairs, there were 13 988 children who were alive at 1 year of age and have been followed with longitudinal clinic- and questionnaire-based assessments (11-13). At the time of analysis, 8927 unrelated individuals of European ancestry had genotyping data available. Detailed characteristics for the ALSPAC cohort and ascertainment of all variables used in analyses are reported in the Supplemental Materials (14). Ethical approval for the study was obtained from the ALSPAC Ethics and Law Committee and the Local Research Ethics Committees, and informed consent for the use of all data collected was obtained from participants following the recommendations of the ALSPAC Ethics and Law Committee at the time.

The collection, data processing, and characteristics of reproductive health data, including repeated assessments of menstrual cycle characteristics, have been previously described in detail (3, 15). Table S1 summarizes the ALSPAC variables used in this study and their sources, including menstrual cycle variables and assessments of features of hyperandrogenism at each time point from approximately age 8 years 1 month to 24 years, along with corresponding hormonal contraception and pregnancy variables (3, 14, 15).

Reproductive health questionnaires were assessed for completeness, and individuals were considered lost to follow-up if they did not provide a response to any reproductive questionnaire. Additional details are provided in the Supplemental Materials (14). For classification of menstrual cycles, individuals were considered to have incomplete data if information on menstrual cycle length, age at menarche, and/or hormonal contraception use were not available. For classification of hyperandrogenism, individuals were considered to have incomplete data if both data on biochemical hyperandrogenism (total testosterone and sex hormone-binding globulin [SHBG] laboratory measurements) and the hirsutism questionnaire were not available. Participants with premature ovarian insufficiency, defined as reporting that periods stopped because of surgery, chemotherapy or radiation therapy, or for no apparent reason or menopause were excluded (Table S1) (3, 14). Data on the use of additional medications used to treat PMOS, including metformin and spironolactone, were not available at the relevant assessment time points.

### Criteria for irregular menses

Menstrual cycle regularity was assessed using repeated questionnaire data on menarche status, usual menstrual cycle length, and hormonal contraception use, which were collected annually from ages 8 to 17 years and at additional assessments in late adolescence and young adulthood (17.5, 19.5, 21, and 24 years) as previously described (3, 15, 16). Briefly, menstrual cycle regularity was defined based on reported usual menstrual cycle length and cycle regularity among participants who had reached menarche and who were not using hormonal contraception at the time of assessment. Detailed descriptions of questionnaire wording, variable processing, and time-point-specific measures are provided in the Supplemental Materials (14).

Consistent with the 2025 international evidence-based recommendations for PMOS in adolescents, we defined irregular menses as (1) onset of menarche after 15 years of age, (2) from 1 to <3 years postmenarche, mean or usual cycle length <21 days or >45 days, and/or (3) from 3 or more years postmenarche, mean or usual cycle length <21 days or >35 days. We also assessed for the guideline criterion of any 1 cycle >90 days occurring at least 1 year postmenarche; this criterion was included to mirror the clinical recommendations and was overlapping rather than mutually exclusive with the age-specific cycle-length definitions above. Age at menarche was determined based on reported age of first menstrual period and was required to assess menstrual cycle regularity, as classification depended on time since menarche (Table S1) (3, 14, 15, 17). Participants were classified as having irregular menses if they met 1 or more of the above criteria at any time point. Questionnaire responses were excluded if there was contraceptive use within 1 year prior to completion of the questionnaire. Responses were also excluded if the timing of pregnancy overlapped with the time period during which menses were classified as irregular.

### Criteria for hyperandrogenism

Total testosterone and SHBG were measured at ∼15.5 years of age (Table S1) (14). The free-androgen index (FAI), a measure of androgen bioavailability, was calculated by dividing the total testosterone level (nmol/L) by the SHBG level (nmol/L) and multiplying by 100 (18). Biochemical hyperandrogenism was defined by a total testosterone and/or FAI >2 standard deviations above the mean for females in the ALSPAC cohort. Because biochemical hyperandrogenism cannot be reliably assessed in individuals using hormonal contraception, we excluded participants who reported contraceptive use within the prior year. Participants who were pregnant at the time of laboratory assessment were also excluded.

Clinical hyperandrogenism was assessed using questionnaire data at 19.5 years (Table S1) (14). Modified Ferriman–Gallwey (mFG) scores were calculated from participant responses on the presence and amount of unwanted/excess body hair in 9 regions of the body (upper lip, chin, chest, upper back, lower back, upper abdomen, lower abdomen, legs, and arms (19)). As the 2025 international evidence-based recommendations for PMOS in adolescents defined a mFG score of ≥4-6 as indicating hirsutism, we defined clinical hyperandrogenism by a score of ≥4 (6). Participants who were pregnant at the time of assessment were excluded because pregnancy can influence hirsutism (20).

Participants were classified as having hyperandrogenism if any available clinical or biochemical assessment met criteria for hyperandrogenism. In our primary analysis, participants were considered to not have hyperandrogenism if data were available for at least one assessment of hyperandrogenism (biochemical or clinical) and no available assessments met criteria for hyperandrogenism. In our secondary sensitivity analysis to assess for potential misclassification from incomplete phenotyping, participants were considered to not have hyperandrogenism if data were available for both biochemical and clinical hyperandrogenism and none of the assessments met criteria for hyperandrogenism.

### Classification of PMOS and at risk for PMOS

**Figure 1:**
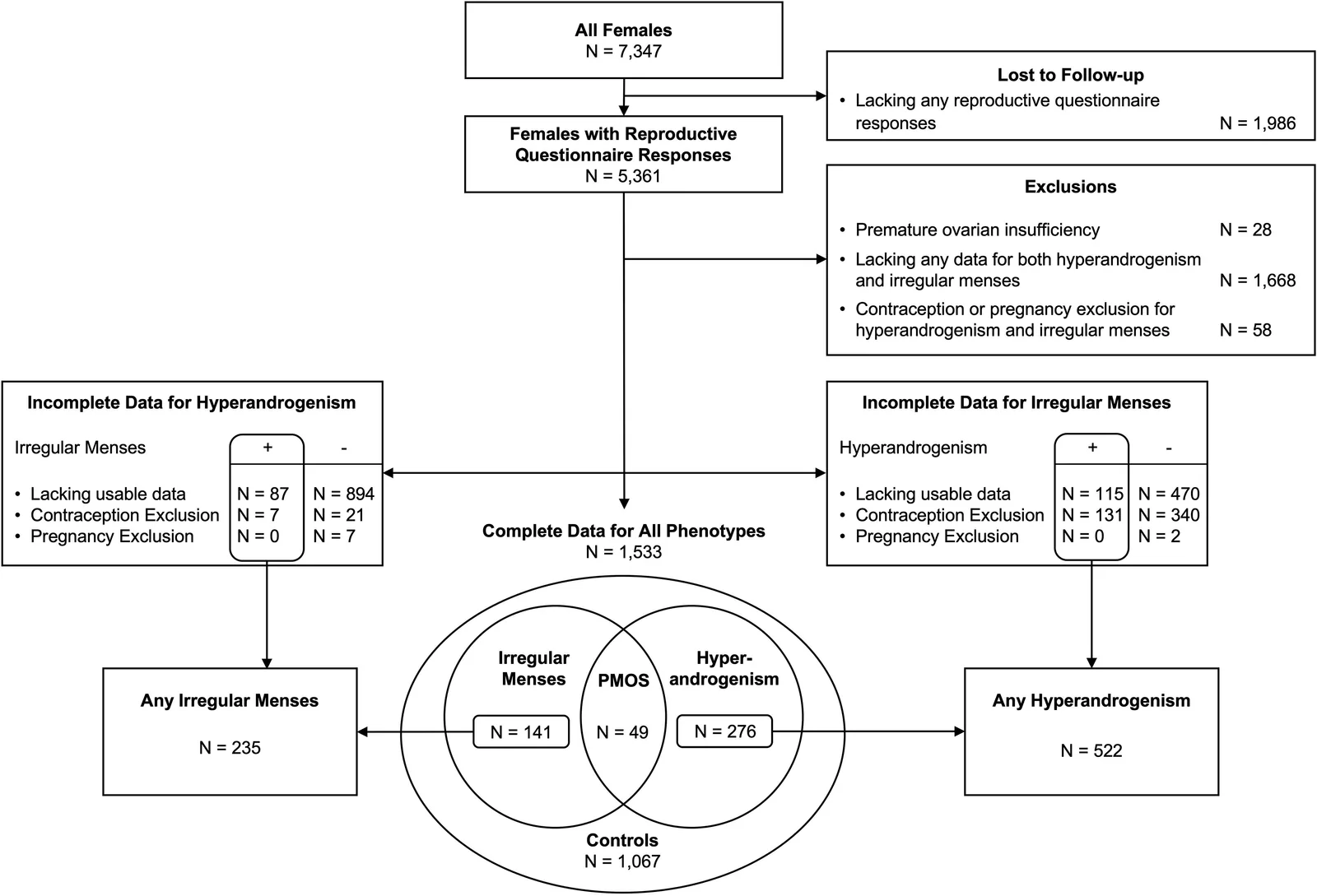
Flowchart of classification of “PMOS” and “at risk for PMOS” in individuals assessed in adolescence and young adulthood in ALSPAC. Calculations of the prevalence of the “PMOS” and “at risk for PMOS” categories were based on those with complete data for all phenotypes. Abbreviation: PMOS, polyendocrine metabolic ovarian syndrome.

PMOS was defined as meeting the criteria for both hyperandrogenism and irregular menses (6). For prevalence estimates, “at risk for PMOS” was defined according to the 2025 international evidence-based recommendations for PMOS in adolescents for those with sufficient data to determine the absence of the other feature (6). Individuals were only classified as controls (ie, not having PMOS or being “at risk for PMOS”) if they responded to the reproductive questionnaire, had sufficient data to evaluate menstrual regularity and hyperandrogenism, and did not meet criteria for PMOS or “at risk for PMOS” (Fig. 1).

For the primary PMOS polygenic score (PGS) analysis, we used the broader categories to incorporate all available longitudinal phenotyping. “Apparently isolated hyperandrogenism” included participants with any hyperandrogenism, even when data on menstrual regularity were incomplete, whereas “apparently isolated irregular menses” included participants with irregular menses, even when data on hyperandrogenism were incomplete. Participants meeting criteria for PMOS were excluded from both groups. We also conducted a secondary PMOS PGS sensitivity analysis restricted to participants with complete data on clinical hyperandrogenism, biochemical hyperandrogenism, and menstrual regularity. In this analysis, participants classified as “at risk for PMOS” had 1 feature with confirmed absence of the other, and controls had neither feature.

### PMOS PGS calculation

Polygenic score for PMOS were calculated using summary statistics from the largest GWAS meta-analysis for PMOS in women of European ancestry (10). PRS-CS software was used to generate a PGS for PMOS using a Bayesian approach that adjusts for linkage disequilibrium by using a reference panel and weighs the effect size of each variant by the strength of its association in GWAS, which allows for inclusion of all variants irrespective of the genome-wide significant threshold (21). This method for calculating PMOS PGS has been shown to predict PMOS as defined by self-report and/or ICD-9/10 codes and is strongly associated with PMOS in adult women in the UK Biobank and further validated in the Estonian Biobank as previously described (22). Our PMOS PGS included 1 119 009 genetic variants and was scaled to a mean of 0 and an SD of 1 to facilitate interpretation.

### Statistical analysis

Logistic regression was used to compare the PMOS PGS between controls and (1) the individuals meeting the criteria for PMOS, (2) the individuals meeting only the hyperandrogenism criteria, classified as “apparently isolated hyperandrogenism” (even if there were insufficient data to evaluate for irregular menses), and (3) the individuals meeting only the irregular menses criteria, classified as “apparently isolated irregular menses” (even if there were insufficient data to evaluate for hyperandrogenism). To adjust for potential population stratification (ie, differences in allele frequencies between subpopulations), we adjusted for the first 10 genotype-derived principal components of ancestry in all analyses.

To address potential misclassification from incomplete phenotyping, we conducted a sensitivity analysis restricted to participants with complete data to assess clinical hyperandrogenism, biochemical hyperandrogenism, and menstrual irregularity. For this analysis, we compared the PMOS PGS between controls and (1) individuals meeting criteria for PMOS, (2) individuals meeting only the hyperandrogenism criteria for “at risk for PMOS,” and (3) individuals meeting only the irregular menses criteria for “at risk for PMOS.”

To evaluate for potential bias arising from the exclusion of individuals with missing data and who reported hormonal contraception use, we conducted 2 secondary analyses. First, we tested for associations between the PMOS PGS and odds of being lost to follow-up as well as odds of having incomplete data for the assessment of irregular menses and/or hyperandrogenism. Second, we tested for associations between the PMOS PGS and odds of contraceptive use, including use to specifically regulate menses and use for other reasons.

## Results

### Prevalence of PMOS and “at risk for PMOS”

Of the 7347 females, 5361 (73%) completed at least 1 reproductive questionnaire and 1533 (21%) had sufficient data to classify both menstrual regularity and hyperandrogenism. Among these 1533 individuals, 49 (3%) met criteria for both irregular menses and hyperandrogenism and were classified as having PMOS. An additional 417 (27%) met criteria for either irregular menses or hyperandrogenism and were classified as “at risk for PMOS”: 276 (18%) met the hyperandrogenism criteria and 141 (9%) met the irregular menses criteria (Fig. 1). The remaining 1067 (70%) individuals did not meet either set of criteria and were classified as controls (eg, not having PMOS or being “at risk for PMOS”) for subsequent analyses (Fig. 1).

### Association between genetic risk for PMOS and “at risk for PMOS”

**Figure 2:**
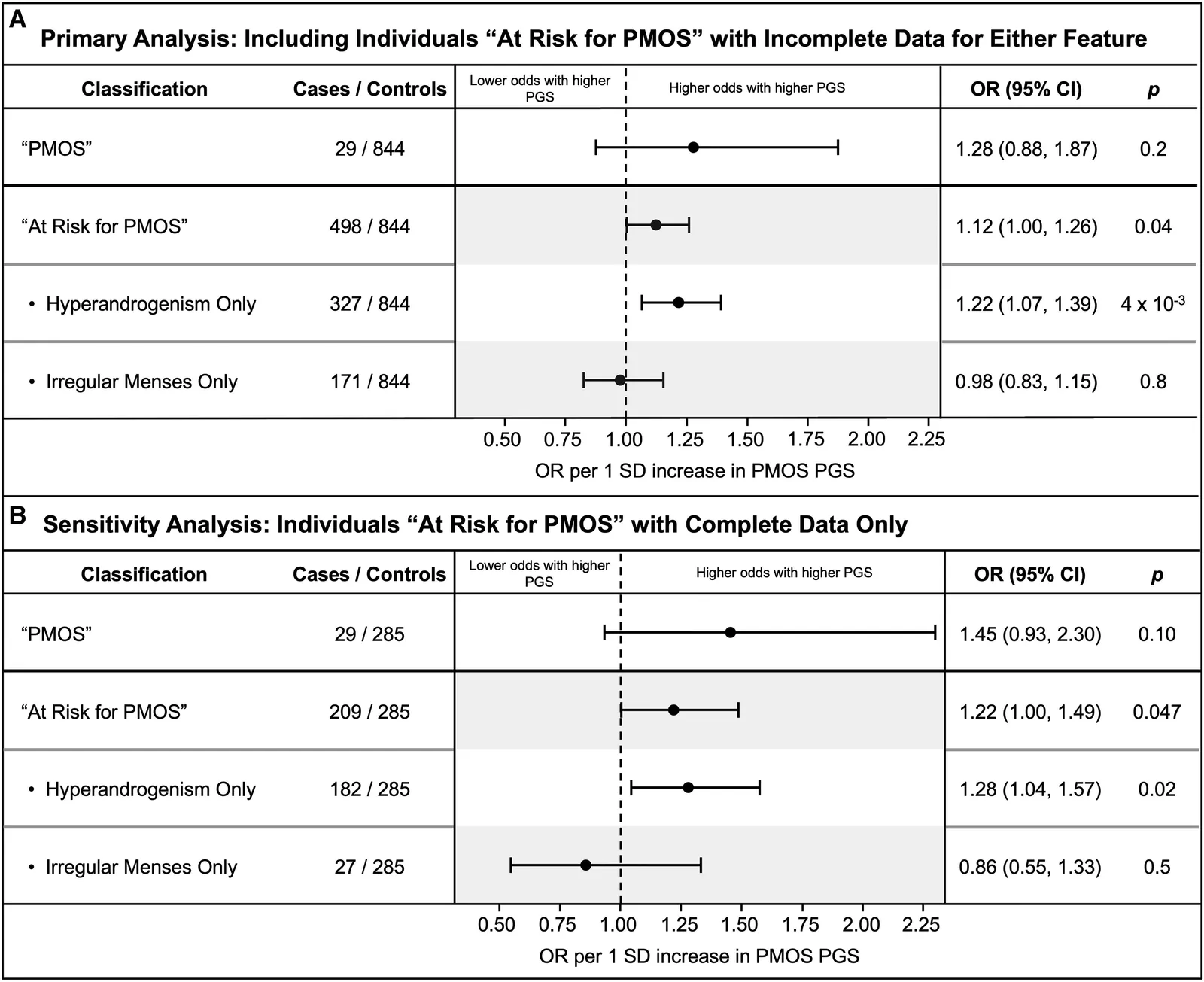
Relationships of PMOS PGS with “PMOS” and “at risk for PMOS.” Associations are shown for PMOS, for the (A) “apparently isolated” or (B) “at risk for PMOS” classification based on either hyperandrogenism or irregular menses, and for the (A) “apparently isolated” or (B) “at risk for PMOS” classification stratified by the defining feature (hyperandrogenism or irregular menses). (A) For the primary analysis, the “apparently isolated” category included any individual who had one feature, including those with insufficient data to evaluate for the other feature. Controls were individuals with (1) sufficient data to assess for menstrual regularity and who did not meet criteria for irregular menses and (2) at least one assessment of hyperandrogenism (biochemical or clinical) and no available assessment meeting criteria for hyperandrogenism. (B) For the secondary sensitivity analysis, the “at risk for PMOS” category included any individual who had 1 feature and who had sufficient data to confirm the absence of the other feature. Specifically, the “at risk for PMOS” by “Irregular Menses Only” classification included any individual who met criteria for irregular menses and who had sufficient data to confirm both absence of clinical and biochemical hyperandrogenism. Controls were individuals with (1) sufficient data to assess for menstrual regularity and who did not meet criteria for irregular menses and (2) both biochemical and clinical assessments for hyperandrogenism and who did not meet criteria for hyperandrogenism. Abbreviations: PMOS, polyendocrine metabolic ovarian syndrome; PGS, polygenic score; OR, odds ratio; CI, confidence interval.

We determined if genetic factors associated with PMOS were associated with “PMOS” and the “at risk for PMOS” classifications by comparing the PMOS PGS between controls (N = 1067) and individuals meeting criteria for PMOS (N = 49) and between controls and 2 broader “apparently isolated hyperandrogenism” and “apparently isolated irregular menses” groups: individuals with any hyperandrogenism but not PMOS (N = 522) and individuals with any irregular menses but not PMOS (N = 235; Fig. 1). Of these total 1873 individuals, we calculated a PGS for PMOS in 1371 individuals who had genotyping data available. We then assessed the relationship between the PMOS PGS and the “apparently isolated hyperandrogenism” and “apparently isolated irregular menses” classifications. A higher PMOS PGS was associated with greater odds of meeting the “apparently isolated hyperandrogenism” classification (OR per 1 SD increase in PGS, 1.22; 95% CI, 1.07-1.39; *P* = 4 × 10^−3^) but not the “apparently isolated irregular menses” classification (OR, 0.98; 95% CI, 0.83-1.15; *P* = .8; Fig. 2A). For comparison, we also assessed the relationship between the PMOS PGS and the “PMOS” classification. Although the association was positive, we did not observe a significant increase in odds of PMOS with increases in the PGS (OR per SD, 1.28; 95% CI, 0.88-1.87; *P* = .2; Fig. 2A). A post hoc power analysis showed that our sample size of 29 cases and 844 controls with genotyping data had only 25% power to demonstrate statistical significance for the observed OR of 1.28 at an α of 0.05 (Fig. 2A).

In addition, we performed sensitivity analyses to assess whether potential misclassification due to incomplete phenotyping could have influenced the observed associations between the PMOS PGS and the “apparently isolated hyperandrogenism/irregular menses” classifications. To assess this, we restricted the analysis to participants with complete data to assess menstrual regularity and both clinical and biochemical hyperandrogenism, which reduced the sample size from 844 to 285 controls, 327 “apparently isolated hyperandrogenism” to 182 individuals “at risk for PMOS” by hyperandrogenism, and 171 “apparently isolated irregular menses” to 27 individuals “at risk for PMOS” by irregular menses. Results were consistent with the primary analyses, with the “at risk for PMOS” by hyperandrogenism associated with higher PMOS PGS (OR per SD, 1.28; 95% CI, 1.04-1.57; *P* = .02), whereas the “at risk for PMOS” by irregular menses was not associated with PMOS PGS (OR per SD, 0.86; 95% CI, 0.55 1.33; *P* = .5; Fig. 2B).

Because hormonal contraception use can affect menstrual regularity and androgen levels, we excluded data on menses and testosterone levels from time points during which individuals reported hormonal contraceptive use. Excluding data associated with hormonal contraceptive use could create bias because hormonal contraception may be used to treat PMOS. To assess this possibility, we tested for associations between the PMOS PGS and odds of hormonal contraception use, including the reported reason for use (eg, menstrual regulation vs other). We did not observe significant associations between genetic risk for PMOS and hormonal contraception use for any reason (*P* all >.3, Fig. S1) (14).

We also assessed potential bias from missing data by testing for associations between the PMOS PGS and odds of being lost to follow-up and having incomplete data for assessment of either menstrual regularity or hyperandrogenism. PMOS genetic risk was not associated with being lost to follow-up, having incomplete menstrual regularity data, or having incomplete hyperandrogenism data (*P* = .09, *P* = .06, *P* = .3, respectively; Fig. S1) (14). Although bias due to missing data cannot be excluded, these findings suggest that exclusions due to missing data were unlikely to have substantially influenced the observed associations between the PMOS PGS and the “at risk for PMOS” classifications.

## Discussion

In this population-based cohort of individuals assessed in adolescence and young adulthood, we observed a PMOS prevalence of 3% and a substantial portion (27%) classified as “at risk for PMOS” by meeting either the hyperandrogenism or irregular menses criteria from the 2025 international evidence-based recommendations for PMOS in adolescents. Notably, a higher PGS for PMOS was associated with increased risk for isolated hyperandrogenism but not with isolated irregular menses. These results suggest that hyperandrogenism in adolescence and young adulthood may more directly reflect inherited liability for PMOS, whereas menstrual irregularity alone may be less specific.

Our observed prevalence of PMOS based on assessments in in adolescence and young adulthood was 3%, lower than the 6% prevalence reported in a recent meta-analysis of global adolescent cohorts (23). Several factors likely contribute to this difference. Prior studies have used heterogeneous and often broader diagnostic criteria for hyperandrogenism and/or irregular menses without specific parameters to define menstrual regularity (23). The ALSPAC cohort also consists predominantly of individuals of European ancestry, which may contribute to a lower prevalence compared with more diverse global cohorts (11). In addition, we considered the possibility that a portion of individuals who were excluded for hormonal contraception use may have specifically initiated hormonal contraception to manage PMOS symptoms, and thus, our estimates could have underestimated the true prevalence. However, we did not identify a significant association between genetic risk for PMOS (used as a proxy for PMOS) and hormonal contraception use (to regulate menses or for other reasons), suggesting that any potential bias resulting from this exclusion did not substantially impact the results.

Multiple factors could reflect the association between genetic risk for PMOS and the hyperandrogenism phenotype but not the irregular menses phenotype. Hyperandrogenism could represent a more specific and earlier manifestation of PMOS pathophysiology, whereas menstrual irregularity during adolescence could arise from several physiologic and environmental factors unrelated to PMOS, including stress, undernutrition, and natural variation in the pace of maturation of the hypothalamic–pituitary–gonadal (HPG) axis. Our PGS for PMOS was derived from GWAS conducted in adult women, and thus, current genetic risk scores for PMOS could also preferentially reflect the androgen-related and other hormonal pathways that manifest or persistent into adulthood. These findings also raise the possibility that the future risk of PMOS may differ by the “at risk for PMOS” phenotype. Given its association with inherited PMOS liability, the hyperandrogenism phenotype may identify adolescents who are more likely to progress to PMOS than those with isolated irregular menses. Longitudinal studies are needed to directly assess the progression to PMOS in these groups.

Several limitations of our study stem from using a longitudinal population-based biobank in which PMOS-related phenotyping was collected across separate study visits over time. Notably, a high portion of individuals had missing reproductive questionnaire data, which could lead to potential bias in our associations with PMOS genetic risk. For example, the menstrual cycle length was self-reported as the participant's usual cycle length over the past 12 months, and participants with very irregular menses may not have been able to provide a response. As a result, we could have excluded some individuals with PMOS or isolated irregular menses, and thus, underestimated the true population prevalence of PMOS and “at risk for PMOS” classifications.

In addition, our approach to include individuals with partially missing data could have led to misclassification; specifically, some individuals classified as “at risk for PMOS” may have met criteria for PMOS if complete phenotyping data had been available, and some individuals classified as controls may have met criteria for “at risk for PMOS.” The sensitivity analysis that we conducted restricted to participants with complete data for biochemical hyperandrogenism, clinical hyperandrogenism, and irregular menses consistently showed associations between the PMOS PGS and “at risk for PMOS” by hyperandrogenism but not isolated irregular menses.

An additional limitation is that biochemical and clinical hyperandrogenism and menstrual cycle regularity were assessed at different developmental time points throughout adolescence and young adulthood. Therefore, these measures reflect these features at the available assessment time points rather than confirming persistence across adolescence and young adulthood. In particular, biochemical and clinical hyperandrogenism were assessed at ages 15.5 and 19.5, respectively, and thus, the classification may not fully capture features that emerged or evolved over time. Consequently, some participants classified as controls or as having isolated irregular menses may have had hyperandrogenism that was not captured by the available assessments. Because ALSPAC did not include a single comprehensive assessment for adolescent PMOS, we integrated all available longitudinal data extending through early adulthood to characterize PMOS-related features across adolescence and into the transition to young adulthood.

Strengths of our study include the size of the population-based ALSPAC cohort, with extensive and repeated measures of clinical, biochemical, and questionnaire-based data across the entire spectrum of adolescence and young adulthood and availability of genetic genotyping data, which allowed for the precise estimates of the “at risk for PMOS” phenotypes and the assessment of links with genetic susceptibility to PMOS.

In conclusion, PMOS prevalence assessed in adolescence and young adulthood using stringent guideline-based criteria was 3%, but over one-fourth met criteria for being “at risk for PMOS.” Genetic risk for PMOS was associated with hyperandrogenism but not isolated irregular menses, emphasizing the key role of hyperandrogenism as an early signal of inherited PMOS liability. Future longitudinal studies of adolescents and young adults at risk for PMOS could characterize progression from at risk states to overt PMOS and integrate genetic, metabolic, and environmental factors to define clinical trajectories that may enable earlier identification and intervention to prevent or delay progression to PMOS and its related conditions.

## Data Availability

Restrictions apply to the availability of all data generated during this study. The informed consent obtained from ALSPAC (Avon Longitudinal Study of Parents and Children) participants does not allow the data to be made available through any third party maintained public repository. Supporting data are available from ALSPAC on request under the approved proposal number, B3581. Full instructions for applying for data access can be found here: http://www.bristol.ac.uk/alspac/researchers/access/.

## References

[1] Joham AE, Norman RJ, Stener-Victorin E, et al Polycystic ovary syndrome. Lancet Diabetes Endocrinol. 2022;10(9):668‐680. 35934017 10.1016/S2213-8587(22)00163-2

[2] Stener-Victorin E, Teede H, Norman RJ, et al Polycystic ovary syndrome. Nat Rev Dis Primers. 2024;10(1):27. 38637590 10.1038/s41572-024-00511-3

[3] Sawyer G, Howe LD, Fraser A, Clayton G, Lawlor DA, Sharp GC. Menstrual cycle features in mothers and daughters in the avon longitudinal study of parents and children (ALSPAC). Wellcome Open Res. 2023;8:386. 37997583 10.12688/wellcomeopenres.19774.3 PMC10665607

[4] Teede HJ, Khomami MB, Morman R, et al Polyendocrine metabolic ovarian syndrome, the new name for polycystic ovary syndrome: a multistep global consensus process. Lancet. 2026;407(10545):2329‐2339. 42119588 10.1016/S0140-6736(26)00717-8

[5] Joham AE, Pena AS. Polycystic ovary syndrome in adolescence. Semin Reprod Med. 2022;40(1-02):e1‐e8. 36096151 10.1055/s-0042-1757138

[6] Pena AS, Witchel SF, Boivin J, et al International evidence-based recommendations for polycystic ovary syndrome in adolescents. BMC Med. 2025;23(1):151. 40069730 10.1186/s12916-025-03901-w PMC11899933

[7] Pena AS, Witchel SF. Update on diagnosis of polycystic ovary syndrome during adolescence. Fertil Steril. 2025;124(5 Pt 2):956‐961. 40945763 10.1016/j.fertnstert.2025.09.006

[8] Kim JJ, Hwang KR, Lee D, Kim S, Choi YM. Adolescents diagnosed with polycystic ovary syndrome under the Rotterdam criteria but not meeting the diagnosis under the updated guideline. Hum Reprod. 2024;39(5):1072‐1077. 38514450 10.1093/humrep/deae042

[9] Teede HJ, Neven ACH, Pena A. Evolution of evidence-based diagnostic criteria in adolescents with polycystic ovary syndrome. Hum Reprod. 2024;39(5):876‐877. 38514447 10.1093/humrep/deae050

[10] Moolhuijsen LME, Zhu J, Mullin BH, et al Genomic analyses implicate hormonal and metabolic dysregulation in polycystic ovary syndrome. Nat Genet. 2026;58(5):1040‐1050. 42026183 10.1038/s41588-026-02543-9 PMC13175888

[11] Boyd A, Golding J, Macleod J, et al Cohort profile: the ‘children of the 90s’–the index offspring of the avon longitudinal study of parents and children. Int J Epidemiol. 2013;42(1):111‐127. 22507743 10.1093/ije/dys064 PMC3600618

[12] Fraser A, Macdonald-Wallis C, Tilling K, et al Cohort profile: the avon longitudinal study of parents and children: ALSPAC mothers cohort. Int J Epidemiol. 2013;42(1):97‐110. 22507742 10.1093/ije/dys066 PMC3600619

[13] Northstone K, Lewcock M, Groom A, et al The avon longitudinal study of parents and children (ALSPAC): an update on the enrolled sample of index children in 2019. Wellcome Open Res. 2019;4:51. 31020050 10.12688/wellcomeopenres.15132.1 PMC6464058

[14] Lingadal, V . Supplemental_Materials_Lingadal_et_al.pdf. figshare. doi: 10.6084/m9.figshare.32337288. Published July 2026.

[15] Elhakeem A, Frysz M, Goncalves Soares A, et al Evaluation and comparison of nine growth and development-based measures of pubertal timing. Commun Med (Lond). 2024;4(1):159. 39112679 10.1038/s43856-024-00580-1 PMC11306255

[16] Christensen KY, Maisonet M, Rubin C, et al Pubertal pathways in girls enrolled in a contemporary British cohort. Int J Pediatr. 2010;2010:329261. 20652082 10.1155/2010/329261 PMC2905723

[17] Golding J, Iles-Caven Y, Northstone K, Fraser A, Heron J. Measures of puberty in the avon longitudinal study of parents and children (ALSPAC) offspring cohort. Wellcome Open Res. 2023;8:453. 38716046 10.12688/wellcomeopenres.19793.2 PMC11075130

[18] Vermeulen A, Verdonck L, Kaufman JM. A critical evaluation of simple methods for the estimation of free testosterone in serum. J Clin Endocrinol Metab. 1999;84(10):3666‐3672. 10523012 10.1210/jcem.84.10.6079

[19] Yildiz BO, Bolour S, Woods K, Moore A, Azziz R. Visually scoring hirsutism. Hum Reprod Update. 2010;16(1):51‐64. 19567450 10.1093/humupd/dmp024 PMC2792145

[20] Yang Y, Han Y, Wang W, et al Assessing new terminal body and facial hair growth during pregnancy: toward developing a simplified visual scoring system for hirsutism. Fertil Steril. 2016;105(2):494‐500. 26616440 10.1016/j.fertnstert.2015.10.036

[21] Ge T, Chen CY, Ni Y, Feng YA, Smoller JW. Polygenic prediction via Bayesian regression and continuous shrinkage priors. Nat Commun. 2019;10(1):1776. 30992449 10.1038/s41467-019-09718-5 PMC6467998

[22] Zhu J, Pujol-Gualdo N, Wittemans LBL, et al Evidence from men for ovary-independent effects of genetic risk factors for polycystic ovary syndrome. J Clin Endocrinol Metab. 2022;107(4):e1577‐e1587. 34969092 10.1210/clinem/dgab838 PMC8947237

[23] Neven ACH, Forslund M, Ranashinha S, et al Prevalence and accurate diagnosis of polycystic ovary syndrome in adolescents across world regions: a systematic review and meta-analysis. Eur J Endocrinol. 2024;191(4):S15‐S27. 39353075 10.1093/ejendo/lvae125

